# One-Year Mortality in Patients Undergoing an Implantable Cardioverter Defibrillator or Cardiac Resynchronization Therapy Pulse Generator Replacement: Identifying Patients at Risk

**DOI:** 10.3390/jcm12175654

**Published:** 2023-08-30

**Authors:** Michelle Feijen, Anastasia D. Egorova, Teresa Kuijken, Marianne Bootsma, Martin J. Schalij, Lieselot van Erven

**Affiliations:** Department of Cardiology, Leiden Heart-Lung Center, Leiden University Medical Center, Albinusdreef 2, 2333 ZA Leiden, The Netherlands; m.feijen@lumc.nl (M.F.); l.van_erven@lumc.nl (L.v.E.)

**Keywords:** pulse generator replacement, ICD, CRT-D, mortality risk, risk prediction

## Abstract

Implantable cardioverter defibrillators (ICDs) significantly contribute to the prevention of sudden cardiac death in selected patients. However, it is essential to identify those who are likely to not have benefit from an ICD and to defer a pulse generator exchange. Easily implementable guidelines for individual risk stratification and decision making are lacking. This study investigates the 1-year mortality of patients who underwent an ICD or cardiac resynchronization therapy with defibrillator function (CRT-D) pulse generator replacement in a contemporary real-world tertiary hospital setting. The cause of death and patient- and procedure-related factors are stratified, and predictive values for 1-year mortality are evaluated. Patients with a follow-up of ≥365 days (or prior mortality) after an ICD or CRT-D exchange at the Leiden University Medical Center from 1 January 2018 until 31 December 2021 were eligible. In total, 588 patients were included (77% male, 69 [60–76] years old, 59% primary prevention, 46% ischemic cardiomyopathy and 37% mildly reduced left ventricular ejection fraction (LVEF)). Patients undergoing a CRT-D replacement or upgrade had a significantly higher 1-year all-cause mortality (10.7% and 11.9%, respectively) compared to patients undergoing ICD (2.8%) exchange (*p* = 0.002). LVEF ≤ 30%, New York Heart Association class ≥ 3, estimated glomerular filtration rate ≤ 30 mL/min/m^2^ and haemoglobin ≤ 7 mmol/L were independently associated with mortality within 1 year after pulse generator replacement. There is a growing need for prospectively validated risk scores to weight individualized risk of mortality with the expected ICD therapy benefit and to support a well-informed, shared decision-making process.

## 1. Introduction

Approximately 50% of all cardiovascular deaths are due to sudden cardiac death (SCD) [1]. The aetiology of SCD strongly depends on age, varying from primarily electrical heart disease and cardiomyopathies in younger patients, to coronary artery disease (CAD) becoming more dominant in the fourth decade and chronic structural disease being most prevalent in older patients [2,3,4]. Implantable cardioverter defibrillators (ICD) continue to significantly contribute to the prevention of SCD and reduction in mortality in selected patient cohorts [2,4,5]. The indication to implant primary and secondary prevention ICDs for patients with ischemic cardiomyopathy is well established and validated [1]. However, since the landmark trials were conducted, heart failure management has drastically improved with cardiac resynchronization therapy (CRT) and novel pharmacological therapies significantly contributing to a reduction in heart failure related hospitalizations and mortality [6,7,8,9,10,11]. Several recent studies questioned the beneficial effects of a primary prevention ICD in patients with non-ischemic cardiomyopathy [5,12,13]. In clinical practice, a personalized risk stratification and prediction of expected ICD benefit for the individual patient remains challenging.

The expected individual ICD benefit should always be weighed against the risks of device-associated complications. These include direct periprocedural complications, as well as the rate of lead failure and risk of device-related infections (pocket infections and/or device endocarditis) [14]. Specifically, pulse generator replacements are associated with a doubled risk of pocket-related re-interventions, and this risk further increases with every consecutive replacement [15]. It is therefore not surprising that the current ESC Guidelines recommend to only implant and/or replace an ICD in patients with an expected good quality of life and expected survival of at least 1 year (class 1C recommendation) [1]. Previous studies indicated that patients with end-stage renal disease, diabetes, atrial fibrillation (AF) and the elderly (octogenarians in particular) are less likely to benefit from a pulse generator exchange [5,16,17]. The World Health Organization reports that the number of persons aged 60 years or above is expected to double to 2 billion by the year 2050, and the number of octogenarians is expected to increase four-fold to 434 million by then [18]. Given the growing complexity of health care systems and the increasing costs, it is essential to timely identify those who are not likely to have any substantial benefit from an ICD and to defer a pulse generator exchange. However, easily implementable guidelines for individual risk stratification and decision making are currently lacking.

The current study investigates the 1-year mortality of patients who underwent an ICD or CRT-D pulse generator replacement in a contemporary real-world tertiary hospital setting. The cause of death and patient- and procedure-related factors are stratified, and predictive values for 1-year mortality are evaluated. Additionally, (extra)cardiac comorbidities and risk factors at time of the pulse generator exchange indicative for mortality are evaluated to determine if a pulse generator exchange could have been avoided based on the known risk factors.

## 2. Materials and Methods

**Figure 1:**
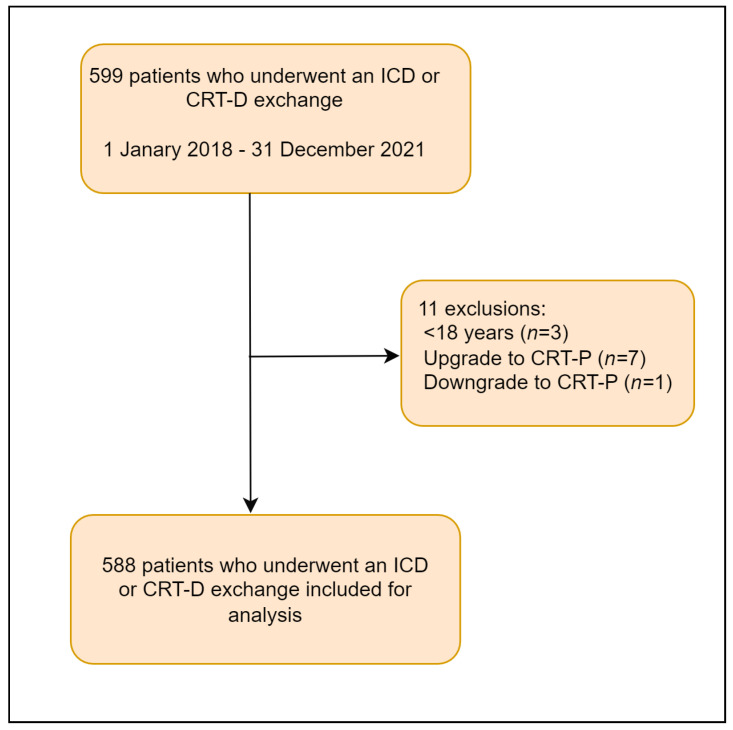
Study flowchart and patient selection.

All patients who underwent an ICD or CRT-D pulse generator exchange (or upgrade) at the Leiden University Medical Center (LUMC) in the period between January 2018 and December 2021 were eligible for inclusion in this retrospective cohort study. Patients had to have completed a follow-up of at least 365 days (or reach the end point of mortality in that time window) to be included for analysis. Patients who underwent an upgrade or downgrade to a cardiac resynchronization therapy pacemaker (CRT-P) and patients < 18 years old were excluded. A study flowchart is depicted in Figure 1.

### 2.1. Data Collection

Demographic and clinical data on risk factors, comorbidities, imaging data, device interrogation and laboratory tests were obtained by retrospective chart review. Clinical data were collected from the hospital electronical patient records—EPD Vision (Leiden, the Netherlands) and HiX (Chipsoft, Amsterdam, The Netherlands). Survival during the study period was assessed via patient chart review and was verified independently with the Dutch personal record database (BRP). In case of mortality, a patient’s records were used to investigate the cause of death. In case of missing or non-conclusive documentation, the general practitioner was contacted to retrieve further information. Furthermore, individual patient risk score to assess the risk of 1-year mortality after pulse generator exchange was calculated according to the previously developed and validated clinical score from Kraaier et al. [19]. This relatively contemporary score was deemed to have the potential to be smoothly implemented in clinical practice, as it is based on generally well established and readily available parameters. The variables used are age ≥ 75 years, left ventricular ejection fraction (LVEF) ≤ 20%, estimated glomerular filtration (MDRD method, eGFR) ≤ 30 mL/min/1.73 m^2^ and history of AF as predictors. Each variable scores 1 point, a risk score ≤1 has a reported expected 1-year mortality risk of 3.4%, a score of 2 has a 1-year mortality risk of 10.9% and a score ≥ 3, 38.9%.

### 2.2. Endpoints

The primary endpoint of the study was 1-year all-cause mortality. The mode of death and patient- and procedure-related factors were stratified, and predictive values for 1-year mortality subsequently evaluated. Additionally, (extra)cardiac comorbidities and known risk factors at the time of the pulse generator exchange indicative for mortality were evaluated to determine if a pulse generator exchange could have been avoided based on the known risk factors.

### 2.3. Statistical Analysis

Normally distributed data were reported as mean ± standard deviation (SD) and non-normally distributed data as median with interquartile range [IQR1–IQR3], unless specifically stated otherwise. Normal distribution was visually assessed and tested with use of the Kolmogorov–Smirnov and the Shapiro–Wilk tests. Normal distributed data were compared using a student t-test, while non-normally distributed data were compared with the Mann–Whitney U test. Proportional differences were compared by applying *χ*^2^ analysis or Fisher’s exact test, as appropriate. Mortality was assessed with the linearized occurrence rate and the Kaplan–Meier method. Cox proportional-hazard models were used to determine the association between the occurrence of death, baseline characteristics and extracardiac comorbidities and estimate the (un)adjusted hazard ratio (HR) and the 95% confidence interval (CI). Covariates were selected based on baseline characteristics and extracardiac comorbidities with a *p*-value < 0.1 in the unadjusted analysis. A stepwise, backward selection method was used to construct a multivariable model. A *p*-value < 0.05 was considered significant. Statistical analysis was performed with IBM SPSS statistics (version 25).

### 2.4. Ethical Statement

The current study was conducted in accordance with the Declaration of Helsinki, applicable local laws and regulations, and the European Data Protection Directive (General Data Protection Regulation). The local ethical committee (Medisch-Ethische Toetingscommissie Leiden Den-Haag Delft) approved the study protocol (2023-020) and waived the need for written informed consent.

## 3. Results

### 3.1. Study Population

In total, 588 patients who underwent an ICD or CRT-D exchange or an upgrade to a CRT-D were included for analysis. The majority of patients were male (*n* = 453, 77%), and the median age was 69 [60–76] years old (Table 1). The indication for the defibrillator was primary prevention in 59% (*n* = 349) of patients, and the underlying aetiology was ischemic cardiomyopathy in most (*n* = 270, 46%) patients, followed by non-ischemic cardiomyopathy (*n* = 242, 41%). Specification of the aetiology is shown in Supplemental Tables S1 and S2. Most patients were in New York Heart Association (NYHA) class I or II, 41% (*n* = 244) and 40% (*n* = 236) respectively. Furthermore, a majority of patients had a mildly (*n* = 215, 37%) or moderately reduced (*n* = 188, 32%) LVEF, and most prevalent valve pathologies were moderate MR (*n* = 127, 29%) and/or moderate TR (164, 28%). AF was diagnosed in 41% of the patients (*n* = 240). Hypertension (*n* = 255, 43%) and chronic renal disfunction (which was defined as an estimated glomerular filtration rate (eGFR) < 60 mL/min/1.73 m^2^ (*n* = 217, 37%) were the most prevalent comorbidities.

### 3.2. Stratification According to Type of Device

Patients were stratified according to the type of pulse generator they received, either ICD exchange (*n* = 286), CRT-D exchange (*n* = 234) or an upgrade to a CRT-D (*n* = 68). Median age was significantly lower in patients undergoing an ICD exchange, 66 years old [54–75] compared to 70 [64–77; 63–77] years old, respectively, in the CRT-D exchange or upgrade groups (*p* < 0.01). Patients who underwent a CRT-D exchange more frequently had a primary prevention ICD indication and were more likely to have a non-ischemic aetiology compared to the ICD-exchange or upgrade patients (*p* = 0.00 for both the variables). Furthermore, patients who underwent an upgrade to a CRT-D or a CRT-D exchange had a higher NYHA class, lower LVEF and more severe MR and/or TR compared to patients undergoing an ICD exchange (*p* < 0.01 for all variables). CRT-D upgrade and exchange patients had a higher prevalence of comorbidities such as AF, diabetes and hypercholesteremia compared to the ICD exchange group (*p* < 0.01). Moreover, these patients have worse renal function (higher creatinine and lower eGFR) and lower haemoglobin (Hb) levels.

In addition, patients who underwent an upgrade to a CRT-D had significantly worse echocardiographic findings (lower LVEF, more severe MR and/or TR) and higher NYHA class (*p* < 0.01) compared to patients that had a CRT-D exchange procedure. Upgrade patients had lower sodium levels and worse renal function (higher creatinine and lower eGFR), and they had more comorbidities (AF and chronic renal failure) compared to CRT-D exchange patients (*p* ≤ 0.01).

### 3.3. Pharmacotherapy

As shown in Table 2, most patients used class II (beta-blockers) or III (sotalol or amiodaron) anti-arrhythmic drugs. Patients with a CRT-D exchange or upgrade used significantly more class II and III and less class IV (diltiazem or verapamil) anti-arrhythmic medication, (*p* = 0.04, *p* = 0.02 and *p* = 0.05, respectively). Additionally, CRT patients used significantly more angiotensin-converting enzyme inhibitors (ACE-I), angiotensin II receptor blockers (ARB) or angiotensin receptor–neprilysin inhibitor (ARNI), more mineral corticoid inhibitors (MRA) and more sodium glucose co-transporter-2 inhibitors (SGLT-2 inhibitors) compared to ICD patients, *p* = 0.00. Interestingly, patients who received a CRT-D upgrade used significantly more ACE/ARB/ARNI, MRA and loop diuretics compared to CRT-D exchange patients, reflecting the worse heart failure status in these patients. In line with the significantly higher percentage of AF in the CRT group, these patients more often used vitamin-K antagonist or direct oral anticoagulants (DOACs), *p* = 0.00. Polypharmacy, defined as ≥5 medications used at the same time for a longer period, was present in the majority of patients (*n* = 461, 78%). Almost all patients with an upgrade to a CRT-D were polypharmacy patients (*n* = 61, 90%), compared to 88% (*n* = 206) in CRT-D exchange and 68% (*n* = 194) in the ICD exchange group, *p* = 0.00.

### 3.4. One-Year Mortality after Pulse Generator Replacement

**Figure 2:**
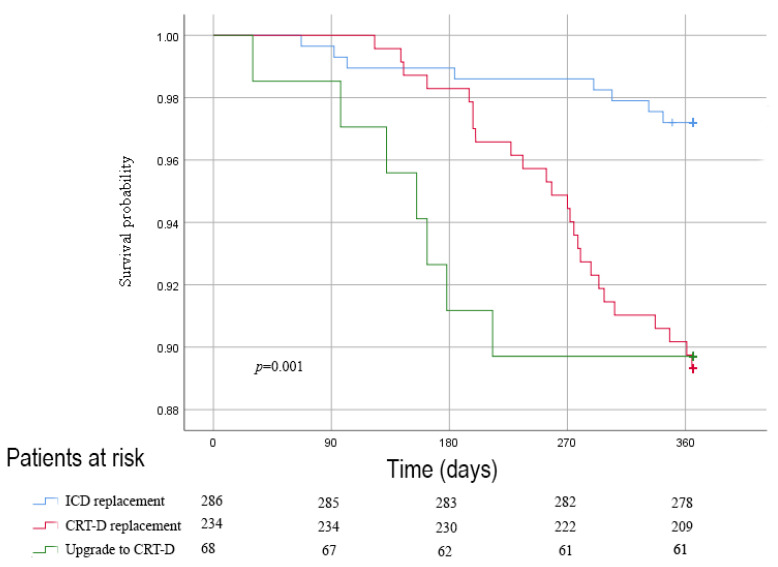
Unadjusted Kaplan–Meier analysis of mortality after pulse generator exchange, stratified by the type of pulse generator exchange (either ICD replacement, CRT-D replacement or an upgrade to a CRT-D).

In the overall cohort, 40 (6.8%) patients died within 1 year after pulse generator exchange procedure, as shown in Table 3. Of these, 53% (*n* = 21) died due to progressive heart failure. No primarily arrhythmia-related deaths were observed. Furthermore, 47% (*n* = 19) died of a non-cardiac cause. Of those, the majority died of cancer (*n* = 6, 32%), followed by pulmonary (*n* = 4, 21%) and infectious diseases (*n* = 4, 21%). Patients who underwent a CRT-D replacement or CRT-D upgrade had a significantly higher 1-year all-cause mortality compared to patients who underwent an ICD exchange, 10.7% (*n* = 25) and 11.9% (*n* = 7), respectively, compared to 2.8% (*n* = 8) (*p* = 0.002, Figure 2). No significant difference in mortality rate was observed between CRT-D exchange and CRT-D upgrade patients (*p* = 0.68). Of interest, even though numerical differences in cause of death can be seen in Table 3, there was no significant difference observed between those with ICD and CRT-D (*p* = 0.35 and *p* = 0.83).

**Figure 3:**
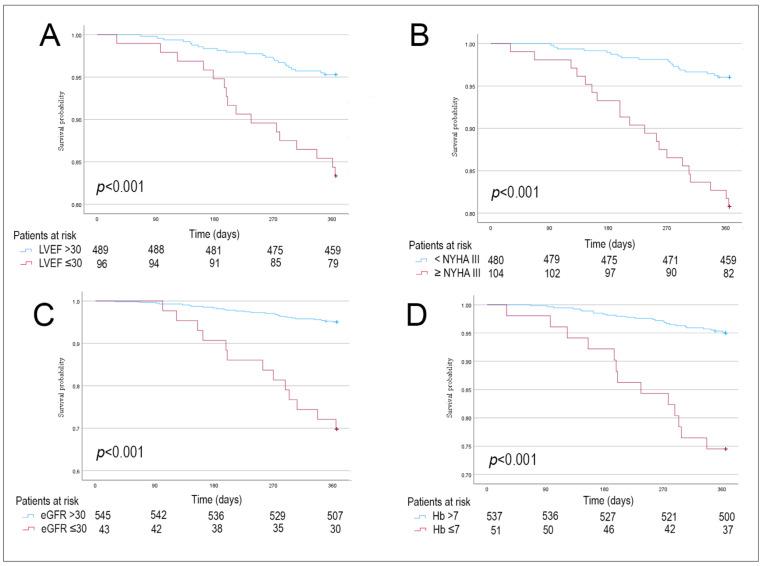
Univariate Kaplan–Meier curves demonstrating the adjusted 1-year mortality risk: (Panel **A**) 1-year mortality risk adjusted for LVEF ≤ 30%; (Panel **B**) 1-year mortality risk adjusted for NYHA class ≥ 3; (Panel **C**) 1-year mortality risk adjusted for eGFR < 30 mL/min/1.73 m; (Panel **D**) 1-year mortality risk adjusted for Hb < 7 mmol/L. Abbreviations: eGFR: estimated glomerular filtration rate; Hb: haemoglobin; LVEF; left ventricular ejection fraction; NYHA: New York Heart Association class.

Significant predictors of 1-year mortality in the univariate analysis included age ≥ 75 years, NYHA class ≥ 3, history of a device infection, AF, the type of pulse generator exchange (either CRT-D replacement or CRT-D upgrade compared to ICD exchange), LVEF ≤ 30%, an increase in estimated systolic pulmonary artery pressure (SPAP, per 1 mm Hg increment), eGFR ≤ 30 mL/min/1.73 m^2^ and Hb ≤ 7 mmol/L (Table 4 and Figure 3).

**Figure 4:**
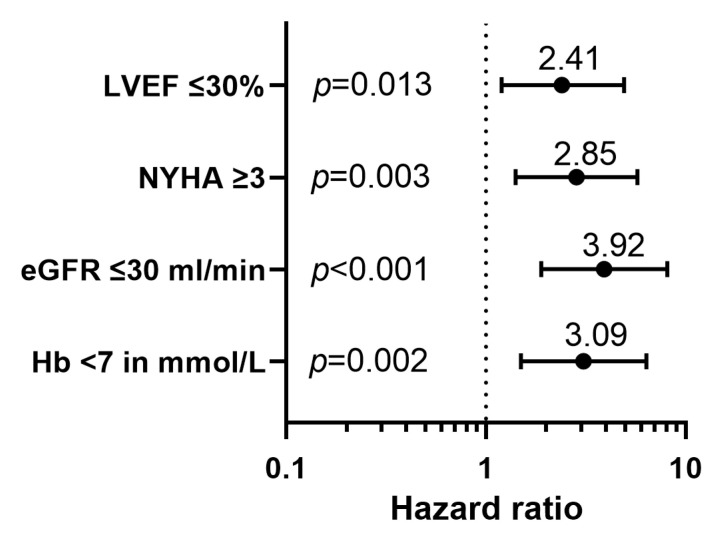
The multivariate survival analysis after correction for type of pulse generator replacement (either ICD replacement, CRT-D replacement or an upgrade to a CRT-D), age, atrial fibrillation, systolic pulmonary artery pressure and number of prescribed medications demonstrates the association between LVEF, NYHA class, Hb and eGFR.

**Figure 5:**
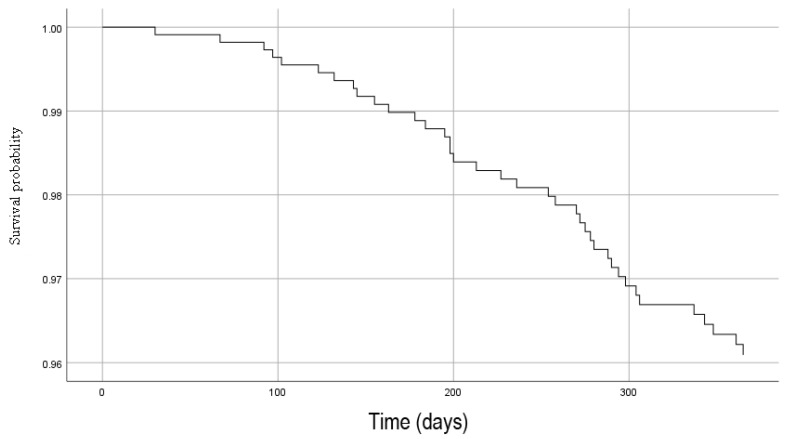
Adjusted Kaplan–Meier survival curves with LVEF, NYHA, eGFR, Hb in the model. Abbreviations: eGFR: estimated glomerular filtration rate; Hb: haemoglobin; LVEF; left ventricular ejection fraction; NYHA: New York Heart Association class.

A multivariate cox proportional hazard model, using stepwise selection of risk factors associated with mortality is depicted in Table 5 and Figure 4. After multivariable adjustment, LVEF ≤ 30% (2.41 [1.20–4.83], *p* = 0.013), NYHA class ≥ 3 (2.85 [1.41–5.74], *p* = 0.003), eGFR ≤ 30 mL/min/1.73 m^2^ (3.92 [1.89–8.11], *p* < 0.001) and Hb ≤ 7 mmol/L (2.83 [1.08–7.43], *p* = 0.002) were independently associated with 1-year mortality. Figure 5 shows the adjusted survival probability.

### 3.5. Validation Risk Score of Kraaier et al.

**Figure 6:**
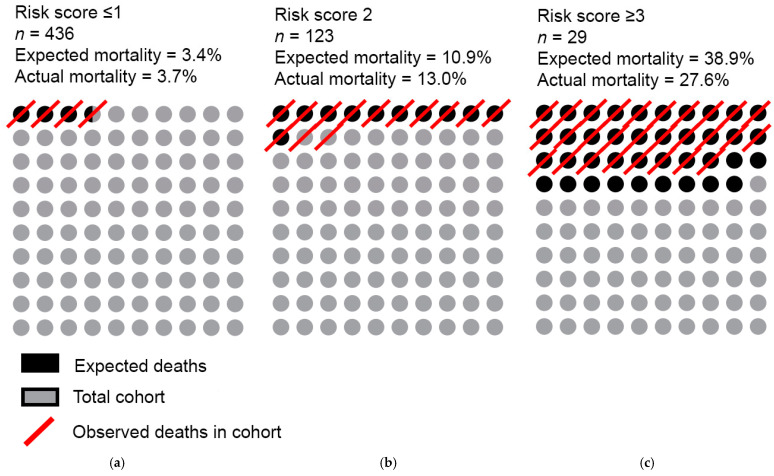
Validation of the risk score developed by Kraaier et al. Panel **a**. shows the patients with risk score 1 (*n* = 436); the expected mortality according to the score is 3.4%, while the actual mortality is 3.7%. Panel **b**. shows the patients with risk score 2 (*n* = 123), expected 10.9% death compared to 13.0% mortality. Panel **c**. shows patients with risk score 3 (*n* = 29); expected death was 38.9%, while the actual mortality was 27.6%.

The risk score developed by Kraaier et al. was used to estimate the expected 1-year mortality of the study population. The expected 1-year mortality with 0 or 1 points was expected to be 3.4% based on literature and entailed 3.7% of current study population (436 patients at risk) (Figure 6). The expected mortality in patients with 2 risk factors was 10.9% according to literature, the actual mortality in this group was 13.0% (out of 123 patients). The expected mortality of patients with risk score ≥ 3 was 38.9%, while in the current cohort only 27.6% (in 29 patients) died within 1 year.

## 4. Discussion

The main finding of the current study is that the 1-year all-cause mortality in patients undergoing an ICD or CRT-D pulse generator exchange was 6.8%. Specifically, patients undergoing a CRT-D replacement or CRT-D upgrade had a significantly higher 1-year all-cause mortality compared to those undergoing an ICD exchange, 10.7% and 11.9%, respectively, compared to 2.8%. LVEF ≤ 30%, NYHA class ≥ 3, eGFR ≤ 30 mL/min/m^2^ and Hb ≤ 7 mmol/L were independently associated with mortality within 1 year after pulse generator replacement. Finally, the score developed by Kraaier et al. was shown to overestimate the expected 1-year mortality in patients with a high-risk score (score ≥ 3, 38.9% expected compared to 26.7% observed mortality) in this contemporary cohort.

According to the 11th World survey on cardiac pacing and implantable cardioverter defibrillators, >200,000 new ICDs were implanted and >105,000 ICDs were replaced (32% of all implants) in 2009 [20]. In current Dutch practice, approximately 6000 ICD and CRT-Ds are implanted annually (data of 2022) [21]. The proportion of pulse generator replacements increases gradually every year, and, in 2018, 25% of all ICD procedures were replacements [21]. The estimated annual expenses for ICD- and CRT-related care are 130 million euros, accounting for >20,000 euros per patient per year. The Dutch National Health Care Institute (Zorginstituut Nederland) systematically assesses healthcare performance and costs, and checks whether diagnostic interventions are being deployed in a patient-orientated, adjudicated and cost-effective manner [21]. The mortality rate of the current cohort (2.8% after ICD replacement and 10.6% after CRT-D replacement and/or upgrade) is in line with the recently published “Appropriate Care” paper that reports a 1-year mortality after ICD replacement of 3.8%, and 8.5% after CRT-D replacement [21]. The national report is based on pooled DRG data, and it is reassuring that a tertiary centre population including patients with congenital heart disease, advanced heart failure and LVADs as destination therapy falls within the expected range, despite the patient complexity.

Kramer et al. previously reported a 1-year mortality after ICD and/or CRT-D replacement in a similar tertiary hospital setting of 9.8% (study 2005–2010) [22]. Another retrospective study (2010–2018) showed a 1-year mortality of 8.3% in patients with a class I indication for an ICD or CRT-D replacement [23]. It is important to note that these studies were performed prior to the amendments of the ESC heart failure guidelines and prior to the implementation of ARNIs and SGLT-2 inhibitors in daily clinical practice, potentially explaining the lower mortality of 6.8% in the current cohort [24].

Current mortality rates call for a better risk stratification of patients undergoing a pulse generator exchange. It is, therefore, of great importance to identify and understand predictors of mortality applicable for individual patients in order to advocate for tailored care and promote educated, shared decision making [25]. Simple and easily implementable risk scores such as the score from Kraaier et al. are promising to aid risk stratification [19]. However, the clinical validation in the current cohort demonstrated an overestimation of the 1-year mortality in the high-risk score group (38.9% based on the model vs. 27.6% in real world data) [19]. These discrepancies raise an important concern from a clinical and ethical point of view—how acceptable is it to defer an individual patient a pulse generator exchange based on concomitant risks, while the 1-year mortality can be substantially lower than expected based on prediction models.

Interestingly, some variables independently associated with 1-year mortality in the current study (LVEF ≤ 30% and eGFR ≤ 30 mL/min/1.73 m^2^) were similar to the previous model, whilst others, i.e., age ≥ 75 years and history of AF were not identified as independent predictors in the current cohort. History of AF is a readily available and easily scored parameter that was associated with 1-year mortality in a univariate analysis; however, after correction in the multivariate model, this prediction value was lost. An important issue with validation is the selection of patients; the current study included primary and secondary prevention patients, and the study from Kraaier et al. only included primary prevention patients [19]. Of interest, in the current cohort, the 1-year mortality after pulse generator exchange did not differ significantly depending on primary vs. secondary prevention ICD indication. However, the literature generally reports secondary prevention ICD patients to have a higher expected mortality; therefore, it is remarkable that the prediction model of Kraaier et al. overestimated mortality in the high-risk patient group (including primary and secondary prevention ICD carriers) [26]. Finally, results from Kraaier et al. were published in 2014, prior to the publication of the new ESC Guidelines and prior to the introduction of ARNI and SGLT-2 inhibitors into daily clinical practice, which might have contributed to the lower observed mortality in the current cohort [24]. Demarchi et al. previously developed a risk stratification model in which permanent AF, eGFR ≤ 30 mL/min/1.73 m^2^, age > 80 years old and a persistent ICD indication were found to be predictors of 1-year mortality [23]. They excluded patients who underwent an upgrade to a CRT-D device, potentially excluding those with worsening heart failure status. Several other studies have previously investigated the risk of death in selected cohorts, such as the FADES score developed by van Rees et al. [27]. The authors demonstrated a post-implant 7% 1-year morality and NYHA ≥ 3, advanced age, DM, LVEF ≤ 25% and a history of smoking were predictors of death in primary prevention ICD carriers without previous appropriate ICD therapy [27]. Additionally, a study of 218 patients that underwent a CRT-D generator exchange (also excluding upgrade procedures), reported a 1-year mortality of 9% [28]. A multivariate logistic regression analysis showed age (>50 per decade), gender (female), Hb (<7.5 mmol/L for female and <8 mmol/L for male), eGFR ≤ 60 mL/min/1.73 m^2^ and prior appropriate shock as risk factors associated with mortality, known as the DARC score. However, the model is not easily embedded into standard care, as it requires the underlying mathematical formula to estimate the mortality risk, and no online calculator tools are currently available. Another study from Jędrzejcyk-Patej et al. compared CRT-D upgrades to de novo CRT-D implants and demonstrated that the all-cause medium-term (4.5 years) mortality was higher in upgrade patients (43.5% vs. 35.5%) [29]. They suggested the CRT scale (creatinine ≥ 150 umol/L, adverse remodelling with left ventricular end systolic diameter ≥ 59 mm and a threshold for NYHA IV) can predict survival following a CRT upgrade [29].

CRT-D therapy is a key component of therapy in heart failure with reduced ejection fraction (HFrEF) and dyssynchrony, on top of pharmacological therapy [24]. It is known that mortality in patients with HFrEF increases with the prolongation of the QRS-complex, and a left bundle branch block (LBB) is further independently associated with increased mortality [30,31]. In addition, CRT therapy is only initiated in patients who are treated with optimal medical therapy for ≥3 months and still experience symptoms of heart failure [24], reflecting the worse heart failure status of this group. Furthermore, many heart failure patients have significant comorbidities reflecting multiorgan impairment, such as renal dysfunction and sleep disorder syndrome. Sleep apnoea itself is associated with a worse prognosis [32]. Remote monitoring rapidly developed during the past decade and with novel algorithms can potentially prompt early diagnosis of sleep apnoea and optimize heart failure treatment [33]. Amongst others, remote monitoring could improve therapeutic compliance and provide long-term monitoring, reducing heart failure-associated admissions [32,34]. Mortality rates for patients with heart failure (regardless of their ejection fraction) remain high. The Olmsted Country cohort reported a 1-year mortality rate of 20% [35]. Therefore, current results emphasize the difficulty in risk stratification and in developing easily implantable risk scores.

Although several models for stratifying patients and predicting individual mortality risks have been proposed, systematic implementation of objective risk stratification prior to a pulse generator exchange is currently lagging behind [36,37]. In order to maximize clinically relevant interventions and anticipate procedure- and ICD-related complications, a comprehensive individualized assessment should be considered mandatory. Given the increasing device longevity, the number of device replacement procedures that a patient will undergo in a lifetime is decreasing. However, patients who survive until each subsequent battery depletion will be (pre)geriatric and are expected to have complex multimorbidity and frailty. Anxiety and depression are inversely correlated with age in ICD patients [38,39]. ICD patients are reported to experience a higher degree of fear of dying compared to pacemaker patients [39]. Despite this, only minority of patients and caregivers structurally discuss end-of-life planning and the potential of disabling the device [38]. This highlights the necessity of a dedicated “pre-exchange” outpatient clinic to obtain a systematic assessment of the patient’s overall health status, the psychological profile and extracardiac comorbidities and to assess the risk of 1-year mortality with the models that are currently at hand. Patient-centred counselling and shared decision making is imperative, during which realistic expectations in terms of quality of life, complications and end-of-life planning can be discussed. However, implementation of educated, shared decision making is more challenging in clinical practice that one might expect [25,40]. An encouraging example of shared decision making is the ‘Dutch ICD decision Aid’ in which patients and physicians are actively involved in the decision process [25].

The current ESC Guidelines do not provide clear guidance for management of ICD patients who no longer have an indication for ICD therapy [41]. A consideration of “CRT super responders” might be to perform a downgrade to a CRT-*p* system [42]. However, technical challenges such as the lack of adapters to correct for the DF-4/IS-1 lead mismatch and thus the requirement of a new right ventricular pacing lead implantation make this option less favourable in clinical practice. Alternatively, programming alternations and abandonment of a device in a non-pacing-dependent patient might be considered. A small cohort study (*n* = 40) recently reported the safety and feasibility of this approach [43]. Lead extraction to facilitate downgrade procedures, avoid venous crowding and maintain MRI compatibility may be considered in selected cases. A recent meta-analysis demonstrated that transvenous lead extraction is a safe and efficacious procedure, and the elderly have similar complication rates compared to younger patients [44]. Current state-of-the-art pacing strategies include conduction system pacing (CSP) to avoid or correct left ventricular dyssynchrony [45,46]. For patients undergoing a pulse generator exchange and/or upgrade with electro-anatomically challenging or failed LV-lead placement, CSP can be considered. However, prospective randomized data comparing the two modalities in the elderly are still to follow.

### Limitations

Several factors should be considered when interpretating the results of the current study. First, this was a single-centre retrospective cohort study that only included patients preselected for an exchange procedure by their referral physician. Patients with overt expected 1-year mortality are therefore expected to have been excluded prior to the identification of this study cohort, introducing potential selection bias. Nevertheless, the findings do reflect the outcomes of a contemporary cohort that is typically encountered by device cardiologists. The setting of a tertiary referral/academic and a non-ischemic cardiomyopathy expertise centre might have included on the one hand younger but, on the other hand, more severely affected patients. It therefore remains to be investigated whether these results are generalizable over a broader range of ICD and CRT-D patients. Finally, the risk factors that have been identified to be independently associated with 1-year mortality after ICD and CRT-D replacement call for prospective multicentre and preferably international validation.

## 5. Conclusions

All-cause 1-year mortality in patients undergoing an ICD or CRT-D pulse generator exchange was 6.8% in this contemporary cohort. Notably, the 1-year mortality in patients undergoing a CRT-D replacement or CRT-D upgrade was significantly higher compared to those undergoing an ICD exchange, 10.7% and 11.9%, respectively, compared to 2.8%. LVEF ≤ 30%, NYHA class ≥ 3, eGFR ≤ 30 mL/min/m^2^ and Hb ≤ 7 mmol/L were independently associated with mortality within 1 year after pulse generator replacement. There is a growing need for prospectively validated risk scores to weight individualized risk of mortality with the expected ICD therapy benefit and to support a well-informed, shared decision-making process.

## Figures and Tables

**Table 1:** Demographic and baseline clinical parameters of the study population (*n* = 588).

	Total Cohort (*n* = 588)	ICD Exchange (*n* = 286)	CRT-D Exchange (*n* = 234)	CRT-D Upgrade (*n* = 68)	*p*-Value ICD vs. CRT-D	*p*-Value CRT Exchange vs. Upgrade
Gender, male (%)	453 (77)	218 (76)	181 (77)	54 (79)	0.75	0.51
Age in years [Q1–Q3]	69 [60–76]	66 [54–75]	70 [64–77]	70 [63–76]	0.00	0.78
BMI in kg/m^2^ [Q1–Q3]	26.5 [24.1–29.7]	26.2 [23.8–29.6]	26.9 [24.7–30.3]	26.4 [24.0–30.0]	0.13	0.97
NYHA-class					0.00	0.00
I, *n* (%)	244 (41)	168 (58)	69 (29)	7 (10)		
II, *n* (%)	236 (40)	89 (31)	114 (49)	33 (49)		
III, *n* (%)	92 (16)	24 (9)	44 (19)	24 (35)		
IV, *n* (%)	12 (2)	3 (1)	5 (2)	4 (6)		
Unavailable, *n* (%)	4 (1)	2 (1)	2 (1)	0 (0)		
**CIED indication**					0.00	0.02
Primary prevention, *n* (%)	349 (59)	146 (51)	166 (71)	37 (54)		
Secondary prevention, *n* (%)	239 (41)	140 (49)	68 (29)	31 (46)		
**Underlying cardiac condition**					0.00	0.414
Ischemic cardiomyopathy, *n* (%)	270 (46)	121 (42)	115 (49)	34 (50)		
Non-ischemic cardiomyopathy, *n* (%)	242 (41)	99 (35)	112 (48)	31 (46)		
Congenital heart disease *n* (%)	29 (5)	23 (8)	5 (2)	1 (2)		
Electrical heart disease *n* (%)	47 (8)	43 (15)	2 (1)	2 (3)	0.11	0.15
**Cardiac history**						
Atrial fibrillation *n* (%)	240 (41)	93 (33)	108 (46)	39 (57)	0.00	0.00
Paroxysmal, *n* (%)	155 (65	67 (72)	59 (55)	29 (74)		
Longstanding/persistent, *n* (%)	85 (35)	26 (28)	49 (45)	10 (26)		
PCI, *n* (%)	183 (31)	81 (28)	70 (30)	32 (47)	0.01	0.00
CABG, *n* (%)	121 (21)	49 (17)	54 (23)	18 (26)	0.10	0.20
Valve surgery, *n* (%)	129 (22)	41 (14)	72 (31)	16 (24)	0.00	0.64
**Echocardiographic findings**						
LVEF					0.00	0.00
Good, *n* (%)	86 (15)	70 (25)	12 (5)	4 (6)		
Mildly reduced, *n* (%)	215 (37)	118 (42)	87 (37)	10 (15)		
Moderately reduced, *n* (%)	188 (32)	77 (27)	86 (37)	25 (37)		
Poor, *n* (%)	96 (16)	18 (6)	50 (21)	28 (42)		
Aortic valve insufficiency (%)					0.15	0.11
No or mild, *n* (%)	535 (91)	260 (91)	217 (93)	58 (85)		
Moderate, *n* (%)	50 (9)	23 (8)	18 (7)	17 (25)		
Severe, *n* (%)	0 (0)	0 (0)	0 (0)	0 (0)		
Mitral valve regurgitation (%)					0.00	0.00
No or mild, *n* (%)	410 (70)	226 (79)	152 (65)	35 (52)		
Moderate, *n* (%)	167 (29)	59 (20)	80 (34)	28 (42)		
Severe, *n* (%)	8 (1)	1 (1)	3 (1)	4 (6)		
Tricuspid valve insufficiency (%)					0.00	0.00
No or mild, *n* (%)	413 (71)	222 (78)	156 (67)	35 (52)		
Moderate, *n* (%)	164 (28)	59 (21)	77 (32)	28 (42)		
Severe, *n* (%)	8 (1)	2 (1)	2 (1)	4 (6)		
sPAP in mm Hg, median [Q1–Q3]	27 [21–34]	26 [20–32]	28 [21–35]	30 [23–38]	0.03	0.11
**Comorbidities and risk** **factors**						
Hypertension, *n* (%)	255 (43)	118 (41)	110 (47)	27 (40)	0.39	0.60
Diabetes mellitus, *n* (%)	126 (20)	40 (20)	59 (25)	17 (25)	0.00	0.25
COPD, *n* (%)	110 (19)	50 (18)	45 (19)	15 (22)	0.64	0.40
Renal function, *n* (%) eGFR in mL/min/1.73 m^2^					0.00	0.00
Normal, eGFR ≥ 60, *n* (%)	350 (59)	202 (71)	121 (52)	27 (40)		
Moderately reduced, eGFR 30–59, *n* (%)	173 (29)	59 (21)	82 (35)	32 (47)		
Severely reduced, eGFR 15–29, *n* (%)	34 (6)	10 (4)	18 (8)	6 (9)		
Kidney failure, eGFR < 15, *n* (%)	9 (2)	2 (1)	7 (3)	0 (0)		
Missing, *n* (%)	22 (4)	13 (4)	6 (1)	3 (4)		
Hypercholesteremia, *n* (%)	181 (31)	69 (24)	91 (39)	21 (31)	0.00	0.89
CVA/TIA, *n* (%)	68 (12)	29 (10)	26 (11)	13 (19)	0.09	0.04
Gastro-intestinal disease, *n* (%)	66 (11)	24 (8)	33 (14)	9 (13)	0.11	0.54
History of malignancy, *n* (%)	78 (13)	34 (12)	34 (15)	10 (15)	0.63	0.70
PADIT-risk score						
Low risk (0–4)	154 (26)	154 (54)	0 (0)	0 (0)	0.00	-
Intermediate risk (5 or 6)	267 (45)	125 (44)	128 (55)	14 (21)	0.00	0.00
High risk (≥7)	167 (28)	7 (3)	14 (21)	54 (79)	0.00	0.00
**Laboratory findings**						
Creatinine in mmol/L, *n* [Q1–Q3]	92 [77–118]	86 [74–105]	99 [80–133]	110 [95–140]	0.00	0.00
eGFR in mL/min/1.73 m^2^, *n* [Q1–Q3]	68 [48–86]	77 [59–89]	62 [40–80]	55 [40–69]	0.00	0.00
Potassium in mmol/L, *n* [Q1–Q3]	4.4 [4.2–4.7]	4.4 [4.2–4.7]	4.4 [4.2–4.7]	4.5 [4.2–4.8]	0.66	0.41

Abbreviations: BMI: body mass index; CABG: coronary artery bypass graft; CIED: cardiac implantable electronic device; COPD: chronic obstructive pulmonary disease; CRT-D: cardiac resynchronization therapy with defibrillator; CVA: cerebrovascular accident; eGFR: estimated glomerular filtration rate; ICD: implantable cardioverter defibrillator; LVEF: left ventricular ejection fraction; NYHA: New York Heart Association class; PADIT score: Prior infection, Age, Depressed renal function, Immunocompromised, Type of procedure; PCI: percutaneous coronary intervention; sPAP: systolic pulmonary artery pressure; TIA: transient ischemic accident.

**Table 2:** Prescribed medication in the study population.

Medication	Total Cohort (*n* = 588)	ICD Exchange (*n* = 286)	CRT-D Exchange (*n* = 234)	CRT-D Upgrade (*n* = 68)	*p*-Value ICD vs. CRT	*p*-Value CRT Exchange vs. Upgrade
Anti-arrhythmic agents (%)						
Class I, *n* (%)	16 (2)	10 (4)	3 (1)	3 (4)	0.19	0.35
Class II, *n* (%)	385 (66)	172 (60)	169 (72)	44 (65)	0.04	0.97
Class III, *n* (%)	190 (32)	80 (28)	81 (35)	29 (43)	0.02	0.51
Class IV, *n* (%)	1 (0)	9 (3)	0 (0)	1 (1)	0.05	0.11
Heart glycosides, *n* (%)	32 (5)	0 (0)	17 (7)	6 (9)	0.15	0.25
Selective sinus node inhibitors, *n* (%)	16 (3)	4 (1)	9 (3)	3 (4)	0.00	0.41
ACE-I/ARB/ARNI, *n* (%)	450 (77)	202 (71)	191 (81)	57 (84)	0.00	0.09
MRA, *n* (%)	204 (35)	52 (18)	117 (50)	35 (51)	0.00	0.00
Loop diuretics, *n* (%)	287 (49)	86 (30)	153 (65)	48 (71)	0.62	0.00
Thiazides, *n* (%)	54 (9)	27 (10)	23 (10)	4 (6)	0.01	0.50
SGLT-2 inhibitor, *n* (%)	11 (2)	1 (0)	7 (3)	3 (4)	0.00	0.12
Anticoagulation/antiplatelet therapy						
Salicylates, *n* (%)	139 (24)	74 (26)	54 (23)	11 (16)	0.11	0.17
P2Y12 blockers, *n* (%)	36 (6)	19 (7)	10 (4)	7 (10)	0.72	0.17
Vitamin K antagonist, *n* (%)	301 (51)	115 (40)	151 (64)	35 (51)	0.00	0.90
DOAC, *n* (%)	52 (9)	22 (8)	13 (6)	17 (25)	0.00	0.00
Polypharmacy *, *n* (%)	461 (78)	194 (68)	206 (88)	61 (90)	0.00	0.01

Abbreviations: ACE-I: angiotensin-converting enzyme inhibitor; ARB: angiotensin II receptor blockers; ARNI: angiotensin receptor–neprilysin inhibitor; DOAC: direct oral anticoagulant; MRA: mineral corticoid inhibitor; SGLT-2 inhibitor: sodium glucose co-transporter-2 inhibitor. * Polypharmacy is defined as ≥5 medications at the same time.

**Table 3:** One-year mortality in the study population, stratified according to the type of exchanged device.

Mortality	1-Year Mortality (*n* = 588)	ICD Exchange (*n* = 268)	CRT-D Exchange (*n* = 234)	CRT-D Upgrade (*n* = 68)	*p*-Value ICD vs. CRT	*p*-Value CRT Exchange vs. Upgrade
All-cause mortality %, (*n*)	6.8% (40)	2.8% (8)	10.7% (25)	11.9% (7)	0.002	0.68
**Cardiac mortality %, (*n*)**	53% (21)	25% (2)	68% (17)	86% (6)	0.35	0.83
Heart failure	100% (21)					
Arrythmia-related death	0					
**Non-cardiac mortality**	47% (19)	75% (6)	32% (8)	14.% (1)		
Cancer	32% (6)					
Renal failure	16% (3)					
Pulmonary disease	21% (4)					
Infectious disease	21% (4)					
Neurological disease	11% (2)					

**Table 4:** Univariate analysis of baseline variables associated with all cause death.

	HR	95% CI	*p*-Value
Gender	0.589	0.25–1.40	0.230
Age ≥ 75 years	2.15	1.15–4.02	**0.017**
BMI in kg/m^2^	1.00	0.95–1.05	0.946
NYHA class ≥ 3	5.92	2.81–9.92	**0.000**
**CIED**			
Primary prevention	0.78	0.41–1.50	0.455
Previous device infection	2.76	1.08–7.04	**0.034**
Number of pulse generator exchanges	1.05	0.697–1.589	0.808
Type of exchange			
ICD			**0.002**
CRT-D exchange	3.94	1.78–8.74	**0.001**
CRT-D upgrade	3.96	1.44–10.93	**0.008**
**Cardiac condition**			
Structural heart disease	1.68	0.41–6.95	0.476
Electrical heart disease	0.31	0.19–4.87	0.401
**Cardiac history**			
Atrial fibrillation	2.22	1.18–4.17	**0.014**
Previous PCI	1.06	0.55–2.06	0.854
Previous CABG	1.29	0.63–2.64	0.484
Previous valve surgery	1.55	0.79–3.05	0.203
**Echocardiographic findings**			
LVEF ≤ 30%	3.76	1.99–7.12	**0.000**
RVF	3.20	0.71–14.02	0.122
sPAP	1.03	1.01–1.09	**0.007**
**Comorbidities and risk factors**			
Diabetes mellitus	2.01	1.07–3.98	**0.039**
COPD	1.10	0.51–2.38	0.815
Hypercholesteremia	1.37	0.72–2.60	0.332
CVA/TIA	1.69	0.75–3.81	0.211
Gastro-intestinal disease	1.41	0.59–3.36	0.436
History of malignancy	0.92	0.36–2.35	0.864
**Laboratory findings**			
eGFR ≤ 30 mL/min/1.73 m^2^	6.91	3.57–13.40	**0.000**
Potassium in mmol/L	3.91	0.57–2.16	0.772
Sodium in mmol/L	0.99	0.97–1.01	0.360
Haemoglobin ≤ 7 mmol/L	5.72	2.95–11.09	**0.000**

Abbreviations: BMI: body mass index; CABG: coronary artery bypass graft; CI: confidence interval; CIED: cardiac implantable electronic device; COPD: chronic obstructive pulmonary disease; CRT-D: cardiac resynchronization therapy with defibrillator; CVA: cerebrovascular accident; eGFR: estimated glomerular filtration rate; HR: hazard ratio; ICD: implantable cardioverter defibrillator; LVEF: left ventricular ejection fraction; NYHA: New York Heart Association class; PCI: percutaneous coronary intervention; RVF: right ventricular function; sPAP: systolic pulmonary artery pressure; TIA: transient ischemic accident.

**Table 5:** Multivariate analysis of baseline variables associated with all cause death.

	HR	95% CI	*p* Value
LVEF ≤ 30%	2.41	1.20–4.83	0.013
NYHA class ≥ 3	2.85	1.41–5.74	0.003
eGFR ≤ 30 mL/min/1.73 m^2^	3.92	1.89–8.11	<0.001
Hb < 7 mmol/L	3.09	1.50–6.37	0.002

Abbreviations: CI: confidence interval; eGFR: estimated glomerular filtration rate; Hb: haemoglobin; HR: hazard ratio; LVEF: left ventricular ejection fraction; NYHA: New York Heart Association class.

## Data Availability

The data presented in this study are available on request from the corresponding author. The data are not publicly available due to privacy restrictions.

## Supplementary Materials

The following supporting information can be downloaded at https://www.mdpi.com/article/10.3390/jcm12175654/s1, Table S1. Type of non ischemic cardiomyopathy and Table S2. Type of electrical heart disease.

## References

[1] Zeppenfeld K., Tfelt-Hansen J., de Riva M., Winkel B.G., Behr E.R., Blom N.A., Charron P., Corrado D., Dagres N., de Chillou C. (2022). 2022 esc guidelines for the management of patients with ventricular arrhythmias and the prevention of sudden cardiac death. Eur. Heart J..

[2] Moss A.J., Hall W.J., Cannom D.S., Daubert J.P., Higgins S.L., Klein H., Levine J.H., Saksena S., Waldo A.L., Wilber D. (1996). Improved survival with an implanted defibrillator in patients with coronary disease at high risk for ventricular arrhythmia. Multicenter automatic defibrillator implantation trial investigators. N. Engl. J. Med..

[3] Kadish A., Dyer A., Daubert J.P., Quigg R., Estes N.A., Anderson K.P., Calkins H., Hoch D., Goldberger J., Shalaby A. (2004). Prophylactic defibrillator implantation in patients with nonischemic dilated cardiomyopathy. N. Engl. J. Med..

[4] Bardy G.H., Lee K.L., Mark D.B., Poole J.E., Packer D.L., Boineau R., Domanski M., Troutman C., Anderson J., Johnson G. (2005). Amiodarone or an implantable cardioverter-defibrillator for congestive heart failure. N. Engl. J. Med..

[5] Jukema J.W., Timal R.J., Rotmans J.I., Hensen L.C.R., Buiten M.S., de Bie M.K., Putter H., Zwinderman A.H., van Erven L., Krol-van Straaten M.J. (2019). Prophylactic use of implantable cardioverter-defibrillators in the prevention of sudden cardiac death in dialysis patients the prospective, randomized, controlled icd2 trial. Circulation.

[6] Yusuf S., Pitt B., Davis C.E., Hood W.B., Cohn J.N. (1992). Effect of enalapril on mortality and the development of heart failure in asymptomatic patients with reduced left ventricular ejection fractions. N. Engl. J. Med..

[7] Pitt B., Zannad F., Remme W.J., Cody R., Castaigne A., Perez A., Palensky J., Wittes J. (1999). The effect of spironolactone on morbidity and mortality in patients with severe heart failure. Randomized aldactone evaluation study investigators. N. Engl. J. Med..

[8] McMurray J.J., Packer M., Desai A.S., Gong J., Lefkowitz M.P., Rizkala A.R., Rouleau J.L., Shi V.C., Solomon S.D., Swedberg K. (2014). Angiotensin-neprilysin inhibition versus enalapril in heart failure. N. Engl. J. Med..

[9] Hjalmarson A., Goldstein S., Fagerberg B., Wedel H., Waagstein F., Kjekshus J., Wikstrand J., El Allaf D., Vítovec J., Aldershvile J. (2000). Effects of controlled-release metoprolol on total mortality, hospitalizations, and well-being in patients with heart failure: The metoprolol cr/xl randomized intervention trial in congestive heart failure (merit-hf). Merit-hf study group. JAMA.

[10] Cleland J.G., Abraham W.T., Linde C., Gold M.R., Young J.B., Daubert J.C., Sherfesee L., Wells G.A., Tang A.S. (2013). An individual patient meta-analysis of five randomized trials assessing the effects of cardiac resynchronization therapy on morbidity and mortality in patients with symptomatic heart failure. Eur. Heart J..

[11] McMurray J.J.V., Solomon S.D., Inzucchi S.E., Køber L., Kosiborod M.N., Martinez F.A., Ponikowski P., Sabatine M.S., Anand I.S., Bělohlávek J. (2019). Dapagliflozin in patients with heart failure and reduced ejection fraction. N. Engl. J. Med..

[12] Køber L., Thune J.J., Nielsen J.C., Haarbo J., Videbæk L., Korup E., Jensen G., Hildebrandt P., Steffensen F.H., Bruun N.E. (2016). Defibrillator implantation in patients with nonischemic systolic heart failure. N. Engl. J. Med..

[13] Disertori M., Quintarelli S., Mazzola S., Favalli V., Narula N., Arbustini E. (2013). The need to modify patient selection to improve the benefits of implantable cardioverter-defibrillator for primary prevention of sudden death in non-ischaemic dilated cardiomyopathy. Europace.

[14] Steckman D.A., Varosy P.D., Parzynski C.S., Masoudi F.A., Curtis J.P., Sauer W.H., Nguyen D.T. (2014). In-hospital complications associated with reoperations of implantable cardioverter defibrillators. Am. J. Cardiol..

[15] Borleffs C.J.W., Thijssen J., de Bie M.K., van Rees J.B., van Welsenes G.H., van Erven L., Bax J.J., Cannegieter S.C., Schalij M.J. (2010). Recurrent implantable cardioverter-defibrillator replacement is associated with an increasing risk of pocket-related complications. Pace.

[16] Junttila M.J., Pelli A., Kenttä T.V., Friede T., Willems R., Bergau L., Malik M., Vandenberk B., Vos M.A., Schmidt G. (2020). Appropriate shocks and mortality in patients with versus without diabetes with prophylactic implantable cardioverter defibrillators. Diabetes Care.

[17] Zabel M., Willems R., Lubinski A., Bauer A., Brugada J., Conen D., Flevari P., Hasenfuß G., Svetlosak M., Huikuri H.V. (2020). Clinical effectiveness of primary prevention implantable cardioverter-defibrillators: Results of the eu-cert-icd controlled multicentre cohort study. Eur. Heart J..

[18] Savelieva I., Fumagalli S., Kenny R.A., Anker S., Benetos A., Boriani G., Bunch J., Dagres N., Dubner S., Fauchier L. (2023). Ehra expert consensus document on the management of arrhythmias in frailty syndrome, endorsed by the heart rhythm society (hrs), asia pacific heart rhythm society (aphrs), latin america heart rhythm society (lahrs), and cardiac arrhythmia society of southern africa (cassa). Europace.

[19] Kraaier K., Scholten M.F., Tijssen J.G., Theuns D.A., Jordaens L.J., Wilde A.A., van Dessel P.F. (2014). Early mortality in prophylactic implantable cardioverter-defibrillator recipients: Development and validation of a clinical risk score. Europace.

[20] Mond H.G., Proclemer A. (2011). The 11th world survey of cardiac pacing and implantable cardioverter-defibrillators: Calendar year 2009—A world society of arrhythmia’s project. Pacing Clin. Electrophysiol..

[21] Van Volksgezondheid M., en Sport W. (2023). Verbetersignalement zinnige zorg implanteerbare cardioverter-defibrillator (icd). Zorginstituut Ned..

[22] Kramer D.B., Kennedy K.F., Spertus J.A., Normand S.L., Noseworthy P.A., Buxton A.E., Josephson M.E., Zimetbaum P.J., Mitchell S.L., Reynolds M.R. (2014). Mortality risk following replacement implantable cardioverter-defibrillator implantation at end of battery life: Results from the ncdr. Heart Rhythm.

[23] Demarchi A., Cornara S., Sanzo A., Savastano S., Petracci B., Vicentini A., Pontillo L., Baldi E., Frigerio L., Astuti M. (2021). Incidence of ventricular arrhythmias and 1-year predictors of mortality in patients treated with implantable cardioverter-defibrillator undergoing generator replacement. J. Am. Heart Assoc..

[24] McDonagh T.A., Metra M., Adamo M., Gardner R.S., Baumbach A., Böhm M., Burri H., Butler J., Čelutkienė J., Chioncel O. (2021). 2021 esc guidelines for the diagnosis and treatment of acute and chronic heart failure. Eur. Heart J..

[25] Yilmaz D., Egorova A.D., Schalij M.J., Spierenburg H.A.M., Verbunt R.A.M., van Erven L. (2022). The development of a decision aid for shared decision making in the dutch implantable cardioverter defibrillator patient population: A novel approach to patient education. Front. Cardiovasc. Med..

[26] Duray G.Z., Schmitt J., Richter S., Israel C.W., Hohnloser S.H. (2009). Arrhythmic death in implantable cardioverter defibrillator patients: A long-term study over a 10 year implantation period. Europace.

[27] van Rees J.B., Borleffs C.J., van Welsenes G.H., van der Velde E.T., Bax J.J., van Erven L., Putter H., van der Bom J.G., Schalij M.J. (2012). Clinical prediction model for death prior to appropriate therapy in primary prevention implantable cardioverter defibrillator patients with ischaemic heart disease: The fades risk score. Heart.

[28] Theuns D., Niazi K., Schaer B.A., Sticherling C., Yap S.C., Caliskan K. (2021). Reassessment of clinical variables in cardiac resynchronization defibrillator patients at the time of first replacement: Death after replacement of crt (darc) score. J. Cardiovasc. Electrophysiol..

[29] Jędrzejczyk-Patej E., Mazurek M., Kotalczyk A., Kowalska W., Konieczny-Kozielska A., Kozielski J., Podolecki T., Szulik M., Sokal A., Kowalski O. (2021). Upgrade from implantable cardioverter-defibrillator vs. De novo implantation of cardiac resynchronization therapy: Long-term outcomes. Europace.

[30] Iuliano S., Fisher S.G., Karasik P.E., Fletcher R.D., Singh S.N. (2002). Qrs duration and mortality in patients with congestive heart failure. Am. Heart J..

[31] Baldasseroni S., Gentile A., Gorini M., Marchionni N., Marini M., Masotti G., Porcu M., Maggioni A.P. (2003). Intraventricular conduction defects in patients with congestive heart failure: Left but not right bundle branch block is an independent predictor of prognosis. A report from the italian network on congestive heart failure (in-chf database). Ital. Heart J..

[32] Mascia G., Perini A.P., Cartei S., Binazzi B., Gigliotti F., Solimene F., Mascioli G., Giaccardi M. (2019). Sleep-disordered breathing and effectiveness of cardiac resynchronization therapy in heart failure patients: Gender differences?. Sleep Med..

[33] Assa S., Vernooy K., van Stipdonk A.M.W. (2023). Cardiovascular implantable electronic devices enabled remote heart failure monitoring; what we have learned and where to go next. J. Cardiovasc. Dev. Dis..

[34] Simantirakis E.N., Schiza S.E., Siafakas N.S., Vardas P.E. (2008). Sleep-disordered breathing in heart failure and the effect of cardiac resynchronization therapy. Europace.

[35] Gerber Y., Weston S.A., Redfield M.M., Chamberlain A.M., Manemann S.M., Jiang R., Killian J.M., Roger V.L. (2015). A contemporary appraisal of the heart failure epidemic in olmsted county, minnesota, 2000 to 2010. JAMA Intern. Med..

[36] Yilmaz D., Egorova A.D., Schalij M.J., van Erven L. (2022). Implantable cardioverter-defibrillators and the older patient: The dutch clinical practice. Eur. J. Cardiovasc. Nurs..

[37] Yilmaz D., van der Heijden A.C., Thijssen J., Schalij M.J., van Erven L. (2017). Patients with an icd remain at risk for painful shocks in last moments of life. J. Am. Coll. Cardiol..

[38] Haugaa K.H., Potpara T.S., Boveda S., Deharo J.C., Chen J., Dobreanu D., Fumagalli S., Lenarczyk R., Madrid A.H., Larsen T.B. (2018). Patients’ knowledge and attitudes regarding living with implantable electronic devices: Results of a multicentre, multinational patient survey conducted by the european heart rhythm association. Europace.

[39] Fumagalli S., Pieragnoli P., Haugaa K.H., Potpara T.S., Rasero L., Ramacciati N., Ricciardi G., Solimene F., Mascia G., Mascioli G. (2019). The influence of age on the psychological profile of patients with cardiac implantable electronic devices: Results from the italian population in a multicenter study conducted by the european heart rhythm association. Aging Clin. Exp. Res..

[40] Elwyn G., Scholl I., Tietbohl C., Mann M., Edwards A.G., Clay C., Légaré F., van der Weijden T., Lewis C.L., Wexler R.M. (2013). “Many miles to go …”: A systematic review of the implementation of patient decision support interventions into routine clinical practice. BMC Med. Inform. Decis. Mak..

[41] Glikson M., Nielsen J.C., Kronborg M.B., Michowitz Y., Auricchio A., Barbash I.M., Barrabés J.A., Boriani G., Braunschweig F., Brignole M. (2021). 2021 esc guidelines on cardiac pacing and cardiac resynchronization therapy. Eur. Heart J..

[42] Frey S.M., Brenner R., Theuns D.A., Al-Shoaibi N., Crawley R.J., Ammann P., Sticherling C., Kühne M., Osswald S., Schaer B. (2023). Follow-up of crt-d patients downgraded to crt-p at the time of generator exchange. Front. Cardiovasc. Med..

[43] Weng W., Theriault-Lauzier P., Birnie D., Redpath C., Golian M., Sadek M.M., Klein A., Ramirez F.D., Davis D.R., Nery P.B. (2022). Should they stay, or should they go: Do we need to remove the old cardiac implantable electronic device if a new system is required on the contralateral side?. Heart Rhythm O^2^.

[44] Lin A.Y., Lupercio F., Ho G., Pollema T., Pretorius V., Birgersdotter-Green U. (2020). Safety and efficacy of cardiovascular implantable electronic device extraction in elderly patients: A meta-analysis and systematic review. Heart Rhythm O^2^.

[45] Senes J., Mascia G., Bottoni N., Oddone D., Donateo P., Grimaldi T., Minneci C., Bertolozzi I., Brignole M., Puggioni E. (2021). Is his-optimized superior to conventional cardiac resynchronization therapy in improving heart failure? Results from a propensity-matched study. Pacing Clin. Electrophysiol..

[46] Vijayaraman P., Herweg B., Ellenbogen K.A., Gajek J. (2019). His-optimized cardiac resynchronization therapy to maximize electrical resynchronization: A feasibility study. Circ. Arrhythm Electrophysiol..

