# Supplementary material for: One-Year Mortality in Patients Undergoing an Implantable Cardioverter Defibrillator or Cardiac Resynchronization Therapy Pulse Generator Replacement: Identifying Patients at Risk
Source: J Clin Med. 2023 Aug 30;12(17):5654. doi: 10.3390/jcm12175654 (PMC10489035; doi:10.3390/jcm12175654)
Supplement: Supplementary file 1 [file jcm-12-05654-s001.zip › jcm-2561740-supplementary.pdf]

Journal of
Clinical Medicine

MDPI

Supplemental Tables

Supplemental Table S1. Type of non-ischemic cardiomyopathy.

Type of Non-Ischemic Cardiomyopathy	Total Cohort (n=240)	ICD Exchange (n=97)	CRT Exchange (n=112)	CRT Upgrade (n=31)
Dilated, n (%)	115 (48)	26 (27)	76 (68)	13 (42)
Hypertrophic, n (%)	52 (22)	40 (41)	7 (6)	3 (10)
Sarcoid, n (%)	6 (3)	2 (2)	1 (1)	3 (10)
Amyloid, n (%)	1 (0)	0 (0)	0 (0)	1 (3)
ARVC, n (%)	11 (5)	9 (9)	2 (2)	0 (0)
Toxic, n (%)	11 (5)	3 (3)	7 (6)	1 (3)
LMNA, n (%)	4 (2)	0 (0)	1 (1)	3 (10)
Other, n (%)	36 (15)	15 (15)	17 (15)	4 (13)

Supplemental Table S2. Type of electrical heart disease.

Type of Electrical Heart Disease	Total Cohort (n=41)	ICD Exchange (n=37)	CRT Exchange (n=2)	CRT Upgrade (n=2)
Brugada, n (%)	6 (15)	5 (14)	1 (50)	0 (0)
Electrical heart disease *n* (%)	4 (10)	4 (11)	0 (0)	0 (0)
Brugada, n (%)	1 (3)	1 (3)	0 (0)	0 (0)
Idiopathic VF, n (%)	30 (73)	27 (73)	1 (50)	2 (100)
